# Treatment Patterns of Long-Acting Somatostatin Analogs for Neuroendocrine Tumors

**DOI:** 10.36469/001c.89300

**Published:** 2023-12-11

**Authors:** Callisia N. Clarke, Paul Cockrum, Thomas J.R. Beveridge, Michelle Jerry, Donna McMorrow, Anh Thu Tran, Alexandria T. Phan

**Affiliations:** 1 Medical College of Wisconsin, Milwaukee, Wisconsin; 2 Ipsen, Basking Ridge, New Jersey; 3 Merative, Ann Arbor, Michigan

**Keywords:** above-label dose, dose escalation, lanreotide, neuroendocrine tumors, octreotide long-acting release

## Abstract

**Background:** Long-acting somatostatin analog therapy (LA-SSA) is recommended as first-line therapy for treatment of unresectable or metastatic neuroendocrine tumors (NETs). Understanding treatment sequencing and dosing patterns of LA-SSA is essential for clinical decision-making to provide value-based management of NETs.

**Objective:** To describe treatment patterns of LA-SSA among patients with NETs and subgroups with carcinoid syndrome (CS) in the United States.

**Methods:** This retrospective study utilized claims data from MarketScan® databases to identify patients with NETs and newly treated with LA-SSA between January 1, 2015, and October 31, 2020. Patients were stratified by index LA-SSA (lanreotide and octreotide long-acting release [LAR]). Reported 28-day doses were based on claim fields for days’ supply/drug quantity or units of service. Dose escalation was defined as increases in quantity or frequency. Continuous variables, categorical variables, and Kaplan-Meier estimated treatment durations were compared using *t*-tests, chi-square/Fisher’s tests, and log-rank tests, respectively.

**Results:** The study included 241 lanreotide and 521 octreotide LAR patients. Compared with octreotide LAR patients, treatment duration was longer for lanreotide patients (median, 41.3 vs 26.8 months; log-rank *p*=.004). Fewer lanreotide patients received rescue treatment with short-acting octreotide (7.9% vs 14.4%; *p*=.011), and a first (6.2% vs 27.3%) and second dose escalation (0.8% vs 5.2%; both *p*<.05). Among patients with doses reported, fewer lanreotide patients received above-label doses (2.5% [5/202] vs 14.4% [60/416]; *p*<.001). Among patients who ended treatment during follow-up, fewer lanreotide patients transitioned to another LA-SSA (18.9% [17/90] vs 33.6% [92/274]; *p*=.008). Similar treatment patterns were observed in CS subgroups. Results for switched treatment patterns were limited due to insufficient sample sizes.

**Discussion:** Real-world treatment patterns of LA-SSA were assessed using more recent administrative claims data. Compared with octreotide LAR patients, lanreotide patients were more likely to remain longer on initial treatment and starting dose without dose escalations and less likely to use rescue treatment and transition to another LA-SSA after discontinuation of the index treatment.

**Conclusions:** Findings from this claims study suggest a potential clinical benefit of lanreotide in NET management.

## INTRODUCTION

Neuroendocrine tumors (NETs) are rare malignancies of neuroendocrine cells that can develop anywhere in the body but most commonly in the gastrointestinal tract, lungs, and pancreas.^1^ In the United States (US), the estimated prevalence was 171 321 patients in 2014.^1^ For unresectable or metastatic NETs of the gastrointestinal tract or pancreas, long-acting somatostatin analogs (LA-SSAs) are recommended as a first-line therapy.^2–4^ Octreotide long-acting release (LAR; Sandostatin® LAR Depot) requires reconstitution for intramuscular injection at a standard dose of 20 mg to 30 mg every 28 days.^5^ Lanreotide (Somatuline® Depot) is available in a prefilled syringe for deep subcutaneous injection at a standard dose of 120 mg every 28 days for gastroenteropancreatic NET.^6^ With the approval of lanreotide for gastroenteropancreatic NET in December 2014, clinicians and patients may consider sequencing between LA-SSAs^7,8^ or escalating LA-SSA doses to above-label 28-day dose (>30 mg for octreotide LAR and >120 mg for lanreotide)^9,10^ to help achieve optimal management of NET. However, real-world evidence for these treatment patterns are limited for lanreotide^9^ and across LA-SSA groups,^11,12^ focusing mostly on octreotide LAR.^10,13–15^ This study aims to describe the treatment patterns of LA-SSA therapy for both the index treatment and switched treatment, among patients with NET treated with LA-SSAs in a large claims-based, privately insured population in the US. Patients were stratified by LA-SSA agent among all patients and patients with carcinoid syndrome (CS).

## METHODS

### Data Source

Administrative claims data from the Commercial, Medicare and Early View versions of the Merative™ MarketScan® databases were used. The Commercial and Medicare databases contain the inpatient, outpatient, and outpatient prescription drug claims data of employees and their dependents covered under fee-for-service and managed care health plans (in the Commercial database), and retirees with Medicare Advantage/Supplemental (in the Medicare database). Both databases include over 19 million lives in 2021. The Early View database includes all components in the Commercial and Medicare databases but captures fully adjudicated healthcare services incurred up to 30 days before data extraction completion. Database records are de-identified and complied with the Health Insurance Portability and Accountability Act. All study data were obtained using codes from the *International Classification of Diseases, Ninth and Tenth Revisions, Clinical Modification*; Healthcare Common Procedure Coding System; and National Drug Codes.

### Study Design

In this retrospective, observational cohort study, the index date was defined as the earliest LA-SSA claim date between January 2015 (to capture claims for lanreotide, which was approved for gastroenteropancreatic NET in December 2014) and October 2020. Patients were classified in 2 overall cohorts (lanreotide and octreotide LAR) based on the LA-SSA received on the index date (ie, index treatment). Within each cohort, patients were stratified into subgroups with and without CS. The baseline period was defined as 12 months prior to the index date. The follow-up period was variable in length. All patients were followed at least 12 months from the index date to the end of continuous enrollment in the databases or the study end date on October 31, 2021, whichever occurred first.

### Patient Selection

Patients were included if they met the following criteria: at least 1 inpatient claim with a NET diagnosis code in the primary position or at least 2 inpatient or non-diagnostic outpatient claims on different days with a NET diagnosis code in any position between January 1, 2015, and October 31, 2020 (earliest NET claim date = NET diagnosis date); at least 18 years of age on the NET diagnosis date; at least 1 claim for LA-SSAs on or after NET diagnosis date (earliest LA-SSA claim date = index date); and at least 12 months of continuous enrollment with medical and pharmacy benefits during both pre- and post-index periods. Patients were required to have no claims for NET treatment (other than short-acting octreotide) (NET treatment list is found in **Appendix Methods 1**) during the pre-index period (to ensure patients were newly treated for NET) and 30 days following the index date (to ensure patients were on a LA-SSA monotherapy); at least 3 months duration of index treatment (to ensure patients were established on the index LA-SSA); and index claims data with non-missing value for days’ supply and drug quantity (for pharmacy claims), or units of service or paid amount (for medical claims) to support dose escalation analysis. Patients diagnosed with CS were identified by diagnosis codes for CS between the start of pre-index period and the end of variable-length follow-up.

### Study Outcomes

Primary outcomes were treatment patterns during index treatment for all patients. Secondary outcomes were treatment patterns during switched treatment (for patients who tried the other LA-SSA and remained on the switched treatment for ≥3 months during follow-up) and during both index treatment and switched treatment of the subgroups of patients with CS. Treatment patterns included treatment duration; doses at initiation and after first and second subsequent dose escalation, up to two dose escalations; use of above-label 28-day dose (>120 mg of lanreotide and >30 mg of octreotide LAR); use of rescue therapy with short-acting octreotide at any time during treatment; and use of other treatment options for NETs. Treatment duration extended from the LA-SSA initiation to the earliest occurrence of a discontinuation gap of over 60 days, switch to the other LA-SSA, or end of follow-up. Doses were reported as 28-day doses based on the claim fields for days’ supply/drug quantity (outpatient pharmacy claims) or units of service (outpatient medical claims) (**Appendix Methods 2**). A dose escalation (within and above-label dose) was defined as having at least 2 consecutive administrations that reflected an increase in either quantity or administration frequency (**Appendix Methods 3**). Additional measures and outcomes are listed in **Appendix Methods 4**.

### Statistical Analysis

Bivariate analyses were conducted for all study variables and reported by index LA-SSA for the overall cohorts and CS subgroups: continuous measures presented as medians, means, and SD; categorical measures presented as counts and percentages. Statistical tests of significance comparing index LA-SSAs in the overall cohorts and CS subgroups were employed, including 2-sample *t*-tests for continuous variables and chi-square tests/Fisher’s exact tests for categorical variables. To account for differing lengths of follow-up, median durations of index treatment were estimated using the Kaplan-Meier method (with censoring of patients whose follow-up ended prior to the end of index treatment) and compared using the log-rank test. Descriptive analyses and significance tests were conducted using WPS Version 4.1 (World Programming, UK). Kaplan-Meier curves were created using R version 4.1.3 (R Foundation for Statistical Computing, Vienna, Austria).

## RESULTS

### Patient Characteristics of Overall Cohorts and CS Subgroups

Among 762 included patients with NET treated with LA-SSA, 241 (31.6%) were indexed on lanreotide and 521 (68.4%) were indexed on octreotide LAR. **Appendix Figure 1** summarizes the patient selection. Lanreotide patients were significantly younger than octreotide LAR patients (mean age, 56.7 vs 59.3, *p* = .002). Female gender was comparable, with 51.9% on lanreotide and 48.4% on octreotide LAR. Severe mean Charlson Comorbidity Index scores of 8.1 for lanreotide and 7.9 for octreotide LAR were measured. A significant difference in trend of index year distribution was observed (*p* < .001), where lanreotide was used increasingly in recent years and for more than half of patients by 2020 (**Table 1**). As a result, compared with octreotide LAR patients, lanreotide patients had shorter follow-up (mean, 27.9 vs 31.9 months; *p* = .001). Use rates of lanreotide vs octreotide LAR were significantly different by type of NET (*p* = .028), which were similar for metastatic NETs (~27%) and different for both benign (20.7% lanreotide vs 29.2% octreotide LAR) and malignant NETs (52.3% lanreotide vs 43.6% octreotide LAR). The primary tumor location of NET was unknown or unspecified on the NET diagnosis date for most patients in each cohort (~65%), with small intestine being the most common location documented in more than 50% of remaining patients with specified locations (**Table 1**).

**Table 1.** Demographic and Clinical Characteristics

	**All Patients**			**Patients with CS**		
	**Lanreotide (n = 241)**	**Octreotide LAR (n = 521)**	***P* Value^b^**	**Lanreotide (n=91)**	**Octreotide LAR (n=240)**	***P* Value^b^**
**Demographic Characteristics^a^**						
Age						
Mean (SD)	56.7 (10.4)	59.3 (11.0)	.002	55.4 (9.1)	59.6 (10.6)	.001
Min-Max	25-90	28-93		25-75	28-89	
Sex, n (%)						
Male	116 (48.1)	269 (51.6)	.369	40 (44.0)	121 (50.4)	.294
Female	125 (51.9)	252 (48.4)		51 (56.0)	119 (49.6)	
Geographic region,^c^ n (%)						
Northeast	26 (10.8)	74 (14.2)	.003	13 (14.3)	33 (13.8)	.121
North Central	54 (22.4)	172 (33.0)		16 (17.6)	72 (30.0)	
South	133 (55.2)	222 (42.6)		54 (59.3)	113 (47.1)	
West	28 (11.6)	52 (10.0)		8 (8.8)	22 (9.2)	
Unknown	0 (0.0)	1 (0.2)		0 (0.0)	0 (0.0)	
Population density, n (%)						
Urban	205 (85.1)	451 (86.6)	.577	75 (82.4)	203 (84.6)	.631
Rural	36 (14.9)	70 (13.4)		16 (17.6)	37 (15.4)	
Payer, n (%)						
Commercial	195 (80.9)	383 (73.5)	.015	79 (86.8)	174 (72.5)	.006
Medicare Supplemental	45 (18.7)	138 (26.5)		12 (13.2)	66 (27.5)	
Medicare Advantage	1 (0.4)	0 (0.0)		0 (0.0)	0 (0.0)	
Index year, n (%)						
2015	39 (16.2)	164 (31.5)	<.001	18 (19.8)	83 (34.6)	<.001
2016	37 (15.4)	129 (24.8)		16 (17.6)	70 (29.2)	
2017	44 (18.3)	81 (15.5)		13 (14.3)	37 (15.4)	
2018	40 (16.6)	47 (9.0)		20 (22.0)	20 (8.3)	
2019	39 (16.2)	61 (11.7)		15 (16.5)	19 (7.9)	
2020	42 (17.4)	39 (7.5)		9 (9.9)	11 (4.6)	
Duration of follow-up, mo						
Mean (SD)	27.9 (15.0)	31.9 (16.2)	.001	30.3 (16.0)	33.5 (16.8)	.115
Median	22.8	28.0		27.2	29.4	
Reason for end of follow-up, n (%)						
End of continuous enrollment	145 (60.2)	373 (71.6)	.002	52 (57.1)	183 (76.3)	<.001
End of the study period	96 (39.8)	148 (28.4)		39 (42.9)	57 (23.8)	
**Clinical Characteristics**						
Charlson Comorbidity Index,^d^ mean (SD)	8.1 (3.1)	7.9 (3.4)	.379	7.9 (3.2)	7.8 (3.5)	.817
NET diagnosis year, n (%)						
2015	84 (34.9)	256 (49.1)	<.001	33 (36.3)	129 (53.8)	.003
2016	32 (13.3)	93 (17.9)		14 (15.4)	47 (19.6)	
2017	41 (17.0)	68 (13.1)		14 (15.4)	31 (12.9)	
2018	31 (12.9)	42 (8.1)		14 (15.4)	14 (5.8)	
2019	33 (13.7)	47 (9.0)		13 (14.3)	16 (6.7)	
2020	20 (8.3)	15 (2.9)		3 (3.3)	3 (1.3)	
Type of NET,^e^ n (%)						
Benign	50 (20.7)	152 (29.2)	.028	18 (19.8)	69 (28.8)	.213
Malignant	126 (52.3)	227 (43.6)		46 (50.5)	101 (42.1)	
Metastatic	65 (27.0)	142 (27.3)		27 (29.7)	70 (29.2)	
Presence of metastatic disease at NET diagnosis,^f^ n (%)	101 (41.9)	218 (41.8)	.986	40 (44.0)	109 (45.4)	.812
Location of primary tumor,^g^ n (%)						
Pancreas	15 (6.2)	19 (3.6)	.515	2 (2.2)	5 (2.1)	.710
Lung/bronchus	13 (5.4)	33 (6.3)		5 (5.5)	14 (5.8)	
Stomach	4 (1.7)	15 (2.9)		1 (1.1)	8 (3.3)	
Small intestine (duodenum, jejunum, ileum)	48 (19.9)	97 (18.6)		25 (27.5)	58 (24.2)	
Cecum	2 (0.8)	8 (1.5)		0 (0.0)	6 (2.5)	
Appendix	1 (0.4)	1 (0.2)		1 (1.1)	0 (0.0)	
Colon	0 (0.0)	2 (0.4)		0 (0.0)	1 (0.4)	
Rectum	0 (0.0)	5 (1.0)		0 (0.0)	2 (0.8)	
Other/ unspecified location	132 (54.8)	295 (56.6)		48 (52.7)	123 (51.3)	
Secondary tumor (unknown primary tumor site)	26 (10.8)	46 (8.8)		9 (9.9)	23 (9.6)	
Time from NET diagnosis to LA-SSA treatment initiation (mo)						
Mean (SD)	9.6 (13.4)	7.2 (10.0)	.008	8.0 (12.1)	6.6 (8.7)	.246
Median	3.3	2.6		2.4	2.2	
Presence of carcinoid syndrome,^h^ n (%)	91 (37.8)	240 (46.1)	.031	91 (100.0)	240 (100.0)	

^a^Evaluated on the index date.

^b^Calculated using 2-sample *t*-tests for continuous variables and chi-square/Fisher’s exact tests for categorical variables.

^c^US Census regions; determined based on the patient’s postal zip code.

^d^Evaluated during the 12-month pre-index period.

^e^Based on ICD-9-CM and ICD-10-CM NET diagnosis codes on the NET diagnosis date and classified by the severity level (metastatic > malignant > benign).

^f^Based on ICD-9-CM and ICD-10-CM diagnosis codes for any secondary malignancy in the 30 days prior to or on the NET diagnosis date.

^g^Based on ICD-9-CM and ICD-10-CM NET diagnosis codes on the NET diagnosis date and classified by the order of location categories listed.

^h^Based on ICD-9-CM and ICD-10-CM diangosis codes for CS between the start of the 12-month pre-index period and the end of the variable-length follow-up period.

Abbreviations: CS, carcinoid syndrome; LAR, long-acting release; LA-SSA, long-acting somatostatin analog; NET, neuroendocrine tumor

Approximately 43.4% (n = 331) of all patients were diagnosed with CS. Lanreotide patients were less likely to be diagnosed with CS compared with octreotide LAR patients (37.8% [91/241] lanreotide vs 46.1% [240/521] octreotide LAR, *p* = .031). Among CS subgroups, similar observations to the overall cohorts were noted for age, gender, reasons for end of follow-up, index year, CCI score, and primary tumor location; however, the type of NETs was not significantly different (**Table 1**).

### Treatment Patterns During Index Treatment of Overall Cohorts

**Figure 1.**
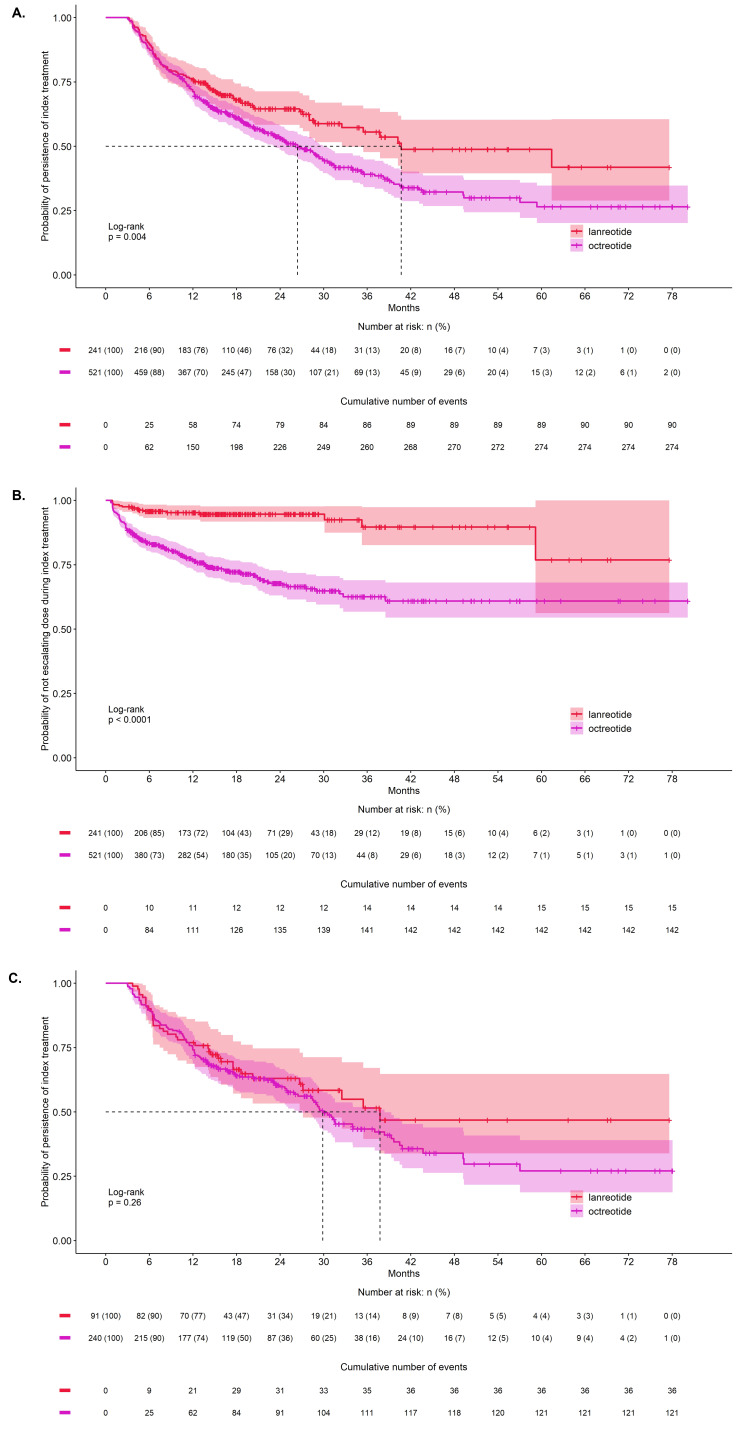
Kaplan-Meier Curves of Time to End of Index Treatment Among All Patients (**A**), Time to First Dose Escalation During Index Treatment Among All Patients (**B**), and Time to End of Index Treatment Among Patients With CS (**C**) Abbreviation: CS, carcinoid syndrome.

Lanreotide patients had longer treatment duration than octreotide LAR patients (log-rank *p* = .004) (**Figure 1A**). After adjusting for differing lengths of follow-up with censoring of patients whose follow-up ended prior to the end of index treatment, the median treatment duration was 41.3 months (95% confidence interval [CI] lower bound: 33.0, upper bound could not be estimated) for lanreotide and 26.8 months (95% CI: 23.6-30.3) for octreotide LAR. Compared with octreotide LAR patients, a lower proportion of lanreotide patients received escalated doses: 6.2% (n = 15) of lanreotide and 27.3% (n = 142) of octreotide LAR patients received first dose escalation (*p* < .001), and 0.8% (n = 2) of lanreotide and 5.2% (n = 27) of octreotide LAR patients received second subsequent dose escalation (*p* = .003) (**Table 2**). Quantity-based dose escalations occurred more often than frequency-based ones: 60.0% (9/15) of lanreotide and 85.9% (122/142) of octreotide LAR patients at first dose escalation, and 100% (2/2) of lanreotide and 70.4% (19/27) of octreotide LAR patients at second dose escalation. Lanreotide patients experienced first dose escalation later than octreotide LAR patients (*p* < .001) (**Figure 1B**). The median time to the first dose escalation was not reported because not enough patients experienced dose escalation.

**Table 2.** Treatment Patterns During Index and Switched Treatment

	**All Patients**		**Patients With CS**	
**During Index Treatment (on Index LA-⁠SSA)**	**Lanreotide**	**Octreotide LAR**	**Lanreotide**	**Octreotide LAR**
	**(n = 241), n (%)**	**(n = 521), n (%)**	**(n = 91), n (%)**	**(n = 240), n (%)**
Reason for end of index treatment, n (%)				
Switch to other LA-SSA	6 (2.5)	39 (7.5)	4 (4.4)	20 (8.3)
Discontinuation of index LA-SSA	84 (34.9)	235 (45.1)	32 (35.2)	101 (42.1)
End of continuous enrollment	94 (39.0)	176 (33.8)	35 (38.5)	87 (36.3)
End of the study period	57 (23.7)	71 (13.6)	20 (22.0)	32 (13.3)
Patients with 1st dose escalation (n, %)	15 (6.2)	142 (27.3)	9 (9.9)	84 (35.0)
Months from initiation to 1st escalation				
Mean (SD)	11.6 (17.2)	7.6 (7.8)	16.7 (20.9)	8.2 (8.8)
Median	4.4	4.3	4.4	4.1
Patients with 2nd dose escalation (n, %)	2 (0.8)	27 (5.2)	2 (2.2)	18 (7.5)
Months from initiation to 2nd escalation				
Mean (SD)	1.9 (0.0)	16.5 (14.1)	1.9 (0.0)	17.1 (17.0)
Median	1.9	11.2	1.9	10.1
Months from 1st to 2nd escalation				
Mean (SD)	1.0 (0.0)	9.1 (11.3)	1.0 (0.0)	8.9 (13.4)
Median	1.0	7.0	1.0	4.3
Use of other NET treatments,^a^ n (%)	78 (32.4)	156 (29.9)	29 (31.9)	85 (35.4)
Short-acting octreotide	19 (7.9)	75 (14.4)	11 (12.1)	41 (17.1)
Targeted therapy	26 (10.8)	51 (9.8)	8 (8.8)	28 (11.7)
Cytotoxic therapy	24 (10.0)	38 (7.3)	6 (6.6)	22 (9.2)
Lutetium ^177^Lu-dotatate	12 (5.0)	28 (5.4)	5 (5.5)	18 (7.5)
Telotristat	16 (6.6)	20 (3.8)	9 (9.9)	16 (6.7)
	**All Patients**		**Patients With CS**	
**During Switched Treatment (Transition to Non-⁠index LA-SSA)^b^**	**Lanreotide to Octreotide LAR**	**Octreotide LAR to Lanreotide**	**Lanreotide to Octreotide LAR**	**Octreotide LAR to Lanreotide**
	**(n = 10), n (%)**	**(n = 67), n (%)**	**(n = 9), n (%)**	**(n = 33), n (%)**
Months from end of index treatment to start of switched treatment				
Mean (SD)	3.8 (10.1)	1.9 (4.2)	4.2 (10.6)	0.9 (2.7)
Median	0.5	0.2	0.5	0.1
Reason for end of switched treatment, n (%)				
Switch back to index LA-SSA	3 (30.0)	2 (3.0)	2 (22.2)	1 (3.0)
Discontinuation of other LA-SSA	3 (30.0)	20 (29.9)	3 (33.3)	11 (33.3)
End of continuous enrollment	3 (30.0)	28 (41.8)	3 (33.3)	13 (39.4)
End of the study period	1 (10.0)	17 (25.4)	1 (11.1)	8 (24.2)
Switched treatment duration^c^ (mo)				
Mean (SD)	15.6 (14.5)	17.0 (14.3)	16.5 (15.0)	18.0 (15.8)
Median	7.3	11.5	7.7	13.0
Patients with 1st dose escalation, n (%)	2 (20.0)	1 (1.5)	2 (22.2)	0 (0.0)
Months from initiation to 1st escalation				
Mean (SD)	13.9 (3.1)	22.3	13.9 (3.1)	
Median	13.9	22.3	13.9	
Patients with 2nd dose escalation, n (%)	0 (0.0)	0 (0.0)	0 (0.0)	0 (0.0)
Use of other NET treatments,^a^ n (%)	6 (60.0)	25 (37.3)	5 (55.6)	19 (57.6)
Short-acting octreotide	4 (40.0)	7 (10.4)	3 (33.3)	5 (15.2)
Targeted therapy	1 (10.0)	10 (14.9)	1 (11.1)	7 (21.2)
Cytotoxic therapy	1 (10.0)	6 (9.0)	1 (11.1)	3 (9.1)
Lutetium ^177^Lu-dotatate	4 (40.0)	3 (4.5)	3 (33.3)	2 (6.1)
Telotristat	2 (20.0)	5 (7.5)	2 (22.2)	4 (12.1)

^a^NET treatments (other than LA-SSA) include targeted therapies (belzutifan, everolimus, sunitinib), cytotoxic chemotherapies (capecitabine, carboplatin, cisplatin, dacarbazine, doxorubicin, etoposide, fluorouracil, ipilimumab, irinotecan, leucovorin, nivolumab, oxaliplatin, pembrolizumab, streptozotocin, temozolomide), interferon alfa-2b (not observed in the study), lutetium 177Lu-dotatate, and telotristat. Patients could have more than one of these other NET treatments (the categories are not mutually exclusive).

^b^Patients could switch immediately (ie, index treatment ended due to switch to the other LA-SSA) or sometimes later after the end of index treatment. Patients were included in switched treatment reporting if they ended index treatment during follow-up, switched to the other LA-SSA after index treatment, remained on that switched treatment for ≥3 months, and had claims data supporting dose escalation analysis during the switched treatment.

^c^Duration of therapy was not adjusted for patients whose follow-up ended prior to the end of LA-SSA treatment. Duration was defined from the start of treatment until the first of a gap of >60 days in treatment, switching to the other LA-SSA, or end of follow-up.

Abbreviations: CS, carcinoid syndrome; LAR, long-acting release; LA-SSA, long-acting somatostatin analog.

**Figure 2.**
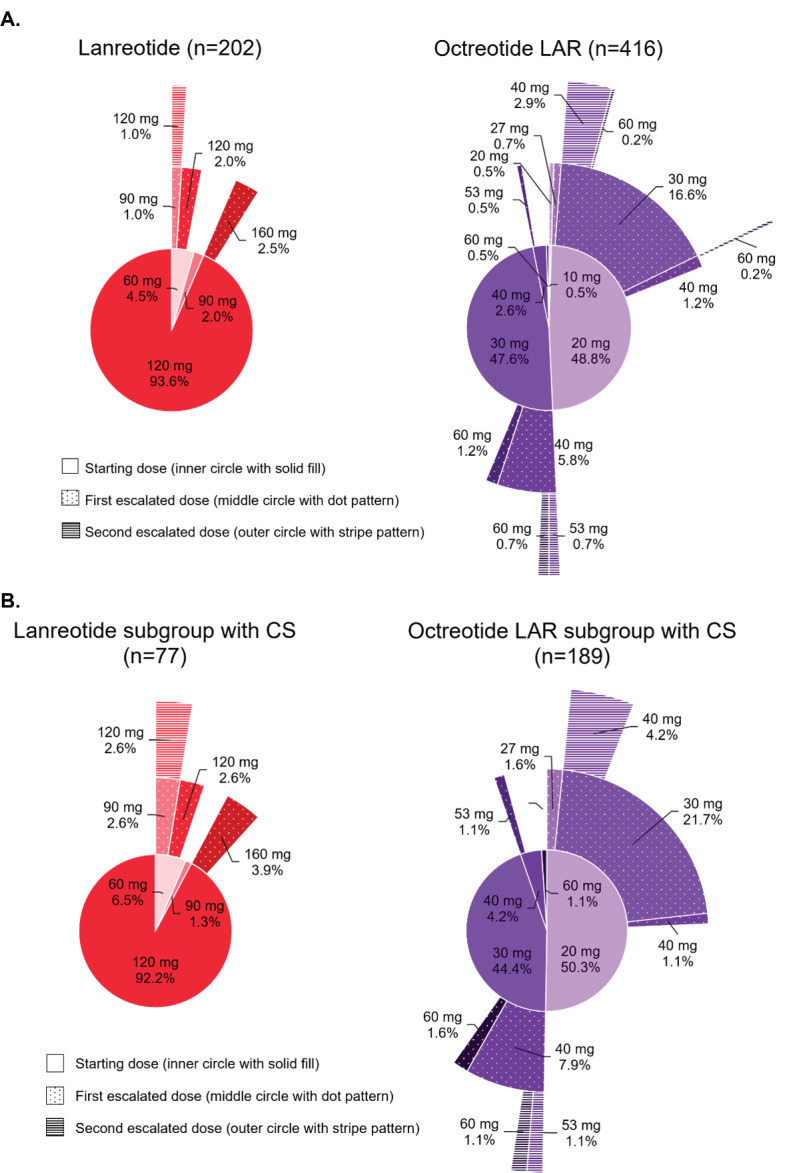
Patterns of Dose Escalation During Index Treatment Among All Patients (**A**) and Among Patients With CS **(B)** Among all patients, doses were reported at treatment initiation, first escalation, and second escalation for 202, 11, and 2 lanreotide patients, and 416, 110, and 20 octreotide LAR patients, respectively.Among patients with CS, doses were reported at treatment initiation, first escalation, and second escalation for 77, 7, and 2 lanreotide patients, and 189, 66, and **1**2 octreotide LAR patients, respectively. The inner, middle, and outer circles show the doses at initiation, after first escalation, and after second dose escalation during the index treatment. The percentages present the proportion of patients with the reported dose among (**A**) the overall cohorts (202 lanreotide and 416 octreotide LAR) and (**B**) the CS subgroups (77 lanreotide and 189 octreotide LAR). For octreotide LAR, non-standard 28-day doses were observed due to frequency-based dose escalations. Doses of 27 mg represented the patients who were on 20 mg every 28 days and increased frequency to every 21 days (20 mg/21*28 = 27 mg), while 53 mg represented the patients who were on 40 mg every 28 days and increased frequency to every 21 days (40 mg/21*28 = 53 mg). Abbreviations: CS, carcinoid syndrome; LAR, long-acting release.

**Figure 3.**
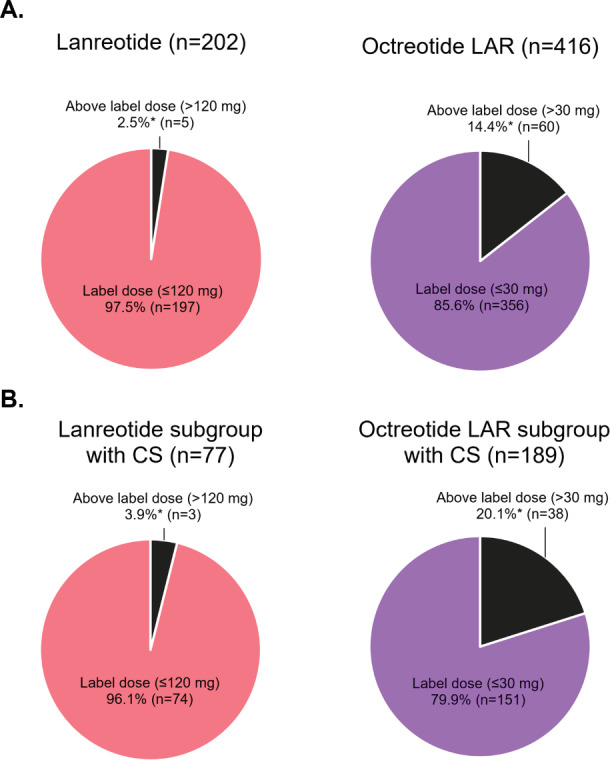
Use of Index LA-SSA Above Label 28-Day Dose During Index Treatment Among All Patients (**A**) and Among Patients With CS (**B**) *p < .001. Abbreviations: CS, carcinoid syndrome; LAR, long-acting release; LA-SSA, long-acting somatostatin analog.

Doses were reported at initiation, first escalation, and second escalation for 202, 11, and 2 lanreotide patients and 416, 110, and 20 octreotide LAR patients, respectively. For the lanreotide cohort, 93.6% of patients received a starting dose of 120 mg. The maximum observed dose was 160 mg and was used for 2.5% of lanreotide patients. Only 2 lanreotide patients, who were also diagnosed with CS, had a second dose escalation. They were initiated at 60 mg then escalated to 90 mg followed by 120 mg, with dose escalations occurring in consecutive 28-day cycles. For octreotide LAR cohort, most patients received a starting dose of 20 mg (48.8%) or 30 mg (47.6%). Patients with a first dose escalation were typically given an additional 10 mg. The maximum observed dose was 60 mg and used for 2.9% of octreotide LAR patients (**Figure 2A**). Among patients with doses reported, 2.5% (5/202) of lanreotide patients received an above label 28-day dose compared with 14.4% (60/416) of octreotide LAR patients (*p* < .001) (**Figure 3A**).

Among all patients, 32.4% of lanreotide and 29.9% of octreotide LAR patients received other treatment options for NETs. Fewer lanreotide patients used rescue treatment with short-acting octreotide at any time during index treatment than octreotide LAR patients (7.9% vs 14.4%, *p* = .011) (**Table 2**).

### Treatment Patterns During Index Treatment of CS Subgroups

Treatment duration did not significantly differ between the CS subgroups (log-rank *p* = .26) (**Figure 1C**). The median treatment duration was 38.3 months (95% CI lower bound: 27.5; upper bound could not be estimated) for lanreotide and 30.3 months (95% CI: 26.9-38.9) for octreotide LAR. Compared with octreotide LAR patients with CS, a lower proportion of lanreotide patients with CS received first dose escalation: 9.9% (n = 9) lanreotide and 35.0% (n = 84) octreotide LAR (*p* < .001). However, the proportion of patients with a second dose escalation was not significantly different among CS subgroups: 2.2% (n = 2) lanreotide and 7.5% (n = 18) octreotide LAR patients (*p* = .071) (**Table 2**).

Doses were reported at initiation, first escalation, and second escalation for 77, 7, and 2 lanreotide patients with CS and 189, 66, and 12 octreotide LAR patients with CS, respectively. Among patients with CS with doses reported, 3.9% (3/77) of lanreotide patients received an above-label 28-day dose compared with 20.1% (38/189) of octreotide LAR patients (*p* < .001) (**Figure 3B**).

Similar observations to the overall cohorts during the index treatment were observed for use of other treatment options for NETs (**Table 2**) and patterns of dose escalation (**Figure 2B**). Use of rescue treatment with short-acting octreotide was not significant different between CS subgroups (*p* = .265) (**Table 2**).

### Treatment Patterns During Switched Treatment of Overall Cohorts and CS Subgroups

Among all patients who ended their index treatment during follow-up, 18.9% (17/90) of lanreotide and 33.6% (92/274) of octreotide LAR patients transitioned immediately or sometime later to the non-index LA-SSA (*p* = .008); 58.8% (10/17) and 72.8% (67/92) of those patients, respectively, remained on the switched treatment for at least 3 months and were included in the switched treatment analysis. **Table 2, Appendix Results 1**, and **Appendix Figure 2** present outcomes during switched treatment of the overall cohorts and CS subgroups.

## DISCUSSION

This retrospective analysis utilized recent administrative claims data (up to October 2021) to assess real-world patterns of LA-SSA dosing and treatment sequences between lanreotide and octreotide LAR in the US. Studies on the treatment patterns of lanreotide are limited due to the more recent approval of lanreotide for gastroenteropancreatic NET in December 2014. Previous studies on LA-SSAs mostly reported the starting vs ending dose,^8,11,15^ the most common escalated dose,^13^ and the proportion of patients with above-label doses.^11,12^ Using outpatient pharmacy and medical claims data, the current study developed a methodology to analyze the dose escalation of LA-SSAs based on increases in doses and frequency. This is the first study that reported up to 2 dose escalations during treatment.

### Treatment Duration

In our study, lanreotide patients remained on the index treatment longer than octreotide LAR patients (41.3 months vs 26.8 months; log-rank *p* < .004), which was similar to the results reported by Harrow et al using a French claims database (31.8 months vs 22.1 months; log-rank *p* < .0001),^7^ indicating higher treatment persistence. The 2 prior US claims studies had reported similar treatment durations of LA-SSAs, including Klink et al (98 days lanreotide vs 228 days octreotide LAR; log-rank p = .61)^12^ and Huynh et al (17.5 months lanreotide vs 19.2 months octreotide LAR; log-rank *p* = .58).^11^ The discrepancy with the prior US claims studies may be due to data availability. Extensive data on lanreotide were available in the French study (8 years during January 2009 to December 2016; lanreotide was approved for NET in France in 2001^16^) and our study (6.8 years during January 2015–October 2021; lanreotide was approved for NET in the US in 2014) compared with the prior US studies (2.0 years during January 2015–December 2016 and 2.9 years during January 2015–November 2017). Treatment duration of LA-SSAs may have been evaluated more accurately with data from a longer study period.

### Dosing Patterns

Dose escalations and use of above-label 28-day doses were less common among lanreotide patients compared with octreotide LAR patients. Although the reasons for dose escalations and use of above-label doses were not documented in claims, other studies have reported the clinical benefits of using up to 60 mg of octreotide LAR every 28 days for improved control of symptoms associated with CS.^10,17^ In the CLARINET FORTE prospective, open-label study of 99 patients with progressive NETs following the standard lanreotide 28-day dose of 120 mg, use of 120 mg every 14 days (ie, 240 mg every 28 days) was associated with stable NETs with no safety concerns.^9^ In addition, dose titration when patients start on a low dose and slowly phase into the maximum tolerated dose of LA-SSA to limit potential adverse effects could not be differentiated from dose escalation due to suboptimal clinical response solely by claims data without clinical data. The only 2 lanreotide patients in this study with a second dose escalation during the index treatment (who started on 60 mg, then escalated to 90 mg, followed by 120 mg at 28-day intervals) may have experienced the dose titration as seen in clinical practice,^18^ rather than dose escalation.

### Transition to the Other LA-SSA

A lower proportion of patients initiated on lanreotide vs octreotide LAR switched to the other LA-SSA after the end of index treatment in this study (18.9% vs 33.6%; *p* = .008), which was similar to observations of the Harrow et al study (11.6% vs 24.8%; *p* < .0001),^7^ the Klink et al study (8.3% vs 17%; *p* = .0243),^12^ and the Huynh et al study (18.0% vs 33.9%; statistical significance not assessed).^11^ However, limited data are available on the clinical benefit and safety of specific LA-SSA sequences. A retrospective medical review of 91 patients who received octreotide LAR followed by lanreotide reported effectiveness associated with lanreotide in stabilizing NETs previously treated with octreotide LAR,^8^ but further prospective studies are warranted to evaluate the effectiveness of transitioning from one LA-SSA to the other for NET management.

### Limitations

The study period included data years shortly after the approval of lanreotide for gastroenteropancreatic NET when most patients were still initiating LA-SSA therapy with octreotide LAR; therefore, the comparisons made in this study may not be representative of current real-world trends because most octreotide LAR patients were indexed in the earlier study years. Dose information of LA-SSAs is sometimes not captured in claims data for office-administered drugs and must therefore be estimated based on paid amount and wholesaler acquisition cost. Because paid amounts can vary widely across provider settings and health plans, these estimations may not reflect the true administered doses. However, since patients often visit the same care setting for their recurring LA-SSA injections, increases in paid amounts for these services likely reflect true dose increases for the dose escalation analysis. Study results for the switched treatment are limited due to the small number of patients who transitioned to the other LA-SSA, partially because of reaching the end of study. The potential for misclassification of NET status or measures are present as patients were identified through claims as opposed to medical records. Claims are subject to data coding limitations and data entry error. This study is limited to individuals with commercial or Medicare coverage and may not be generalizable to patients with other insurance or without health coverage. Due to the observational nature of the study, some of the differences in study outcomes between cohorts may be due to differences in baseline characteristics; therefore, future studies that adjust for differences in baseline characteristics between patients initiating lanreotide vs octreotide LAR are warranted.

## CONCLUSIONS

Findings from this claims study suggest a potential clinical benefit of lanreotide. Compared with octreotide LAR patients, patients with NET who were newly treated with lanreotide were more likely to remain longer on their initial LA-SSA treatment, continue the starting dose without dose escalation for longer, and less likely to use rescue treatment. Study results during the switched treatment are limited due to the insufficient sample size. Additional studies over a longer period are warranted to confirm these important findings and better evaluate the treatment patterns during switched treatment.

### Author Contributions

All authors were involved in study design, analysis and interpretation of the data, drafting and revising the paper, and approving the final version of the manuscript. All authors agree to be accountable for all aspects of the work.

### Competing Interests

P.C. and T.B. are employees of Ipsen. M.J. and A.T. are employees of Merative which received funding from Ipsen to conduct this study. D.M. was an employee of Merative at the time the study was conducted. C.C. and A.P. have nothing to disclose.

### Ethics Approval and Informed Consent

All database records are de-identified and fully compliant with US patient confidentiality requirements, including the Health Insurance Portability and Accountability Act (HIPAA) of 1996. The databases have been evaluated and certified by an independent third party to be in compliance with the HIPAA statistical de-identification standard. The databases were certified to satisfy the conditions set forth in §§ 164.514 (a)-(b)1ii of the HIPAA privacy rule regarding the determination and documentation of statistically de-identified data. Because this study uses only de-identified patient records and does not involve the collection, use, or transmittal of individually identifiable data, the data does not involve human subjects (per the definition of human subjects in the 45 CFR § 46.102(e)). Thus, this study was exempted from Institutional Review Board approval. Data was used under license for this study.

### Meeting Presentation

This study was presented in part at the 2022 NANETS Multidisciplinary NET Medical Symposium in Washington, DC.

### Data Availability

The data that support the findings of this study are available from Merative. Restrictions apply to the availability of these data, which were used under license for this study.

## Supplementary Material

Appendix
