# Supplementary material for: Treatment Patterns of Long-Acting Somatostatin Analogs for Neuroendocrine Tumors
Source: J Health Econ Outcomes Res. 2023 Dec 11;10(2):121–31. doi: 10.36469/001c.89300 (PMC10718524; doi:10.36469/001c.89300)
Supplement: Appendix [file jheor_2023_10_2_89300_188906.pdf]

Journal of Health Economics and Outcomes Research

Online Supplementary Material

Treatment Patterns of Long-Acting Somatostatin Analogs for Neuroendocrine Tumors. *JHEOR*. 2023;10(2):121-131. doi:10.36469/jheor.2023.89300

Appendix Methods 1: Neuroendocrine Tumor (NET) Treatments Included in the Study (Based on Published Standard[1,2] and Clinician Input)

Appendix Methods 2: Dose Calculation Using Data on the Claim Fields

Appendix Methods 3: Definitions of Dose Escalation

Appendix Methods 4: Other Study Outcomes

Appendix Results 1: Outcomes During Switched Treatment

Appendix Figure 1: Patient Selection

Appendix Figure 2: Patterns of Dose Escalation During Switched Treatment Among All Patients (A) and Among Patients With CS (B)

This supplementary material has been provided by the authors to give readers additional information about their work.

This is an open-access article distributed under the terms of the Creative Commons Attribution 4.0 International License (CCBY-4.0). View this license's legal deed at http://creativecommons.org/licenses/by/4.0 and legal code at http://creativecommons.org/licenses/by/4.0/legalcode for more information.

APPENDIX METHODS 1: NEUROENDOCRINE TUMOR TREATMENTS INCLUDED IN THE STUDY (BASED ON PUBLISHED STANDARD[1,2] AND CLINICIAN INPUT)

- Long-acting somatostatin analogs: lanreotide and octreotide long-acting release
- Targeted therapies: belzutifan, everolimus, and sunitinib
- Cytotoxic chemotherapies: capecitabine, carboplatin, cisplatin, dacarbazine, doxorubicin, etoposide, fluorouracil, ipilimumab, irinotecan, leucovorin, nivolumab, oxaliplatin, pembrolizumab, streptozotocin, and temozolomide
- Others: interferon alfa-2b, lutetium ^{177}Lu-dotatate, and telotristat

APPENDIX METHODS 2: DOSE CALCULATION USING DATA ON THE CLAIM FIELDS

The methodology used to calculate the dose of long-acting somatostatin analogs (LA-SSAs) was different for outpatient pharmacy fills (using National Drug Codes [NDC] codes) vs office-administered injections (using Healthcare Common Procedure Coding System [HCPCS] codes).

For LA-SSAs that were dispensed at an outpatient pharmacy, the strength, quantity, and days' supply of LA-SSAs were documented. The 28-day dose was calculated as

[(Strength * Quantity)/Days' Supply] * 28.

For LA-SSAs that were administered at an outpatient office, the administered dose was often reported in the claim field for units of service. If the units of service were missing or invalid, the administered dose was estimated by the paid amount on claim and wholesale acquisition cost (WAC) using the formula:

(Paid Amount/WAC) * Strength Associated With WAC

The WAC of lanreotide 120 mg and octreotide LAR 20 mg were used in the dose estimation as previous studies reported the average price of a standard 28-day dose of LA-SSAs based on these products.[3,4] The WAC applicable as of the date of service was used in the dose calculation to account for the changes in WAC over time. The 28-day dose was calculated as

(Administered Dose/Time Between Injections) * 28

The time between injections was defined as the number of days between the given injection and the prior injection and capped at 28 days (standard duration of clinical benefit for LA-SSAs).

APPENDIX METHODS 3: DEFINITIONS OF DOSE ESCALATION

Patients were identified as having a dose escalation[5] if they had at least 2 consecutive administrations that reflected an increase in either quantity or frequency of administration. If a patient had 2 consecutive frequency-based dose escalations or 2 consecutive quantity-based dose escalations at the same time, the patient was categorized as having had a quantity-based dose escalation. Quantity-based dose escalation was defined as having 2 consecutive doses with an increase of at least 30% over the starting dose. Frequency-based dose escalation was defined as having 2 consecutive injections more often (decreased time between injections using HCPCS claims or reduced day's supply using NDC claims) that resulted in at least a 30% increase of 28-day doses over the index dose. For example, if a patient changes a regimen of 120 mg of lanreotide from every 28 days to every 21 days (increased frequency), the 28-day dose increases by at least 30% from 120 mg to 160 mg (120 mg/21*28). If the frequency of injections increased while the quantity decreased, the patient's 28-day dose may have not reached an increase of at least 30%. This scenario therefore was not considered as a frequency-based dose escalation. For example, if a patient changes a regimen of 120 mg of lanreotide every 28 days to 90 mg (decreased quantity) every 21 days (increased frequency), the 28-day dose remained unchanged at 120 mg (90 mg/21*28).

APPENDIX METHODS 4: OTHER STUDY OUTCOMES

Additional outcomes were reported during the index treatment and switched treatment, including duration from the treatment initiation to the first and second dose escalation, duration between 2 dose escalations, and reasons for end of treatment. Demographic characteristics were reported on the index date. Charlson Comorbidity Index was measured during the pre-index period. NET diagnosis characteristics were described, including year of NET diagnosis, type of NET, presence of metastatic disease, and locations of primary tumor. Time from NET diagnosis date to the initiation of index LA-SSA was measured.

APPENDIX RESULTS 1: OUTCOMES DURING SWITCHED TREATMENT

Among all patients, the median durations of switched treatment (without adjusting for patients whose follow-up ended prior to the end of LA-SSA treatment) were 7.3 months for transition to octreotide LAR and 11.5 months for transition to lanreotide. Patients who transitioned to octreotide LAR were more likely to end the switched treatment by re-initiating the index LA-SSA (30.0%) than

patients who transitioned to lanreotide (3.0%). Rescue treatment with short-acting octreotide was used in 40.0% of patients who transitioned to octreotide LAR and 10.4% of patients who transitioned to lanreotide (**Table 2**). **Appendix Figure 2** represents the patterns of dose escalation during switched treatment. For patients with doses reported, above-label 28-day doses were used in 28.6% (2/7) of patients who transitioned to octreotide LAR, but not used in patients who transitioned to lanreotide.

Among patients with CS who ended their index treatments during follow-up, 30.6% (11/36) of lanreotide and 38.8% (47/121) of octreotide LAR patients transitioned immediately or sometime later to the non-index LA-SSA ($p = 0.366$); of which 81.8% (9/11) and 70.2% (33/47) of those patients, respectively, remained on the switched treatment for at least 3 months and included in the analysis. Rescue treatment was used in 33.3% of patients who transitioned to octreotide LAR and 15.2% of patients who transitioned to lanreotide (Table 2).

Appendix Figure 1. Patient Selection

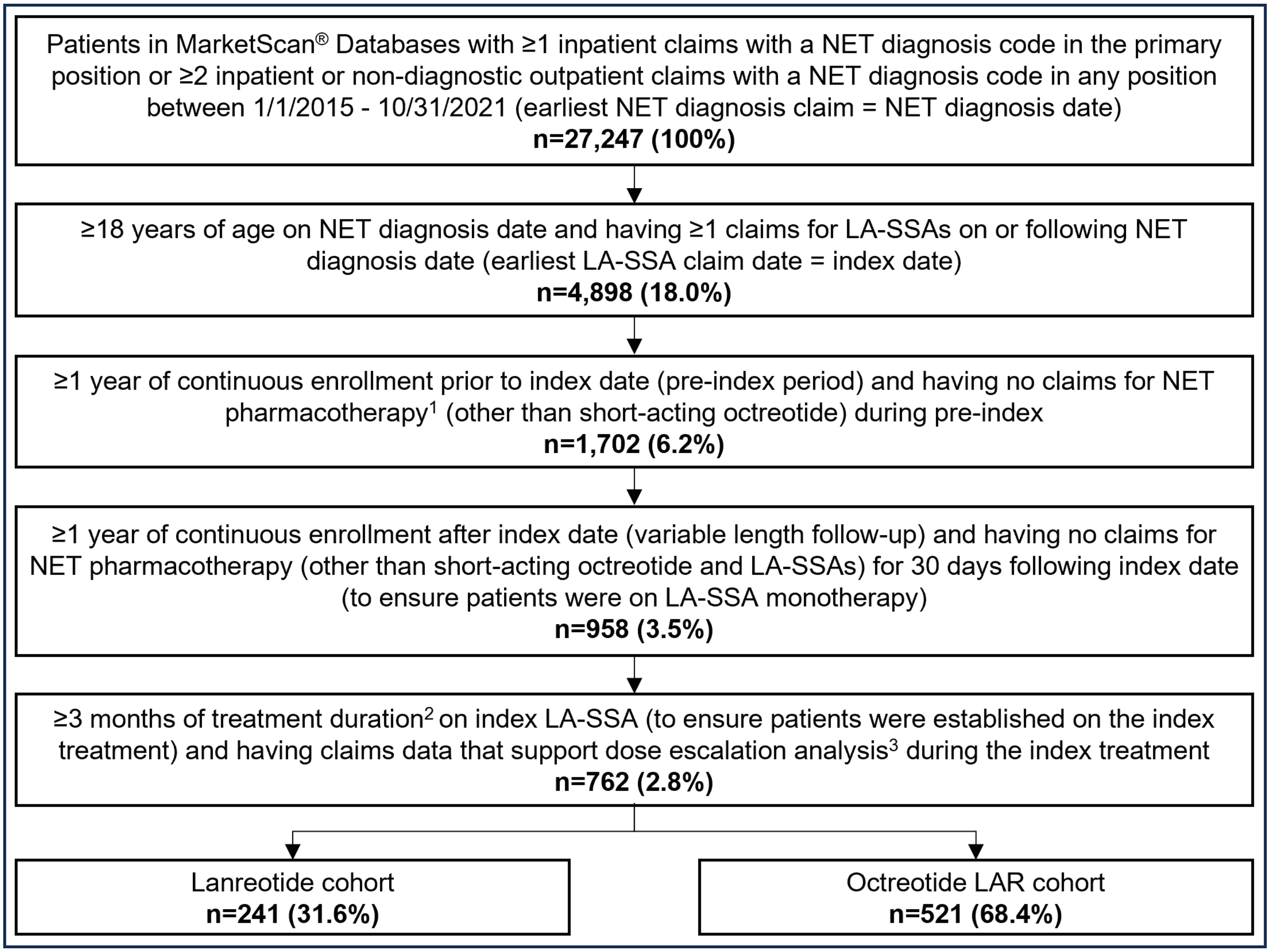

[1]The following treatment options for NETs were included: LA-SSA (lanreotide and octreotide long-acting), targeted therapies (belzutifan, everolimus, sunitinib), cytotoxic chemotherapies (capecitabine, carboplatin, cisplatin, dacarbazine, doxorubicin, etoposide, fluorouracil, ipilimumab, irinotecan, leucovorin, nivolumab, oxaliplatin, pembrolizumab, streptozotocin, temozolomide), and others (interferon alfa-2b, lutetium ^{177}Lu-dotatate, telotristat). Although short-acting octreotide is used to treat NETs, it was not included in patient selection because it is often used prior to and during LA-SSA treatment.

[2]Duration of LA-SSA treatment was measured from the initiation of index LA-SSA until the first of a gap of >60 days in treatment, switching to the other LA-SSA, or end of follow-up.

[3]To support dose escalation analysis, the index claim needed to have non-missing data for days' supply/drug quantity (for pharmacy claims) or units of service or paid amount (for medical claims).

Abbreviations: LAR, long-acting release; LA-SSA, long-acting somatostatin analog; NET, neuroendocrine tumor.

Appendix Figure 2. Patterns of Dose Escalation During Switched Treatment Among All Patients (**A**) and Among Patients With CS (**B**)

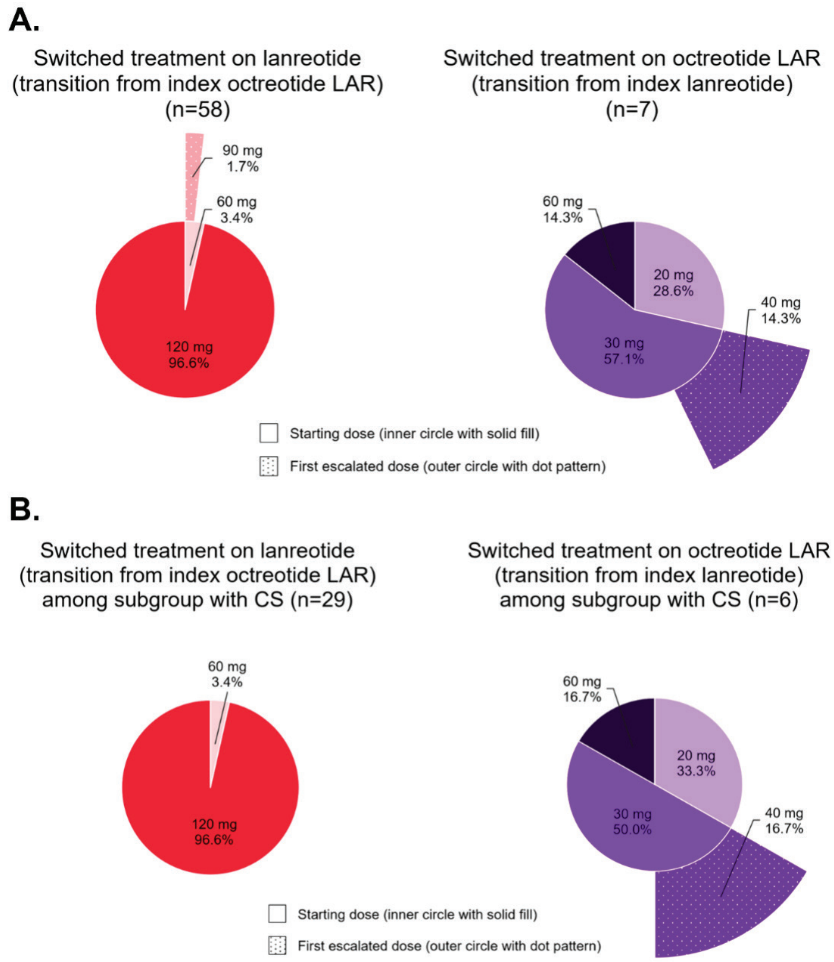

(**A**) Among all patients, doses were reported at treatment initiation and first escalation for 58 patients and 1 patient who transitioned to lanreotide, and 7 patients and 1 patient who transitioned to octreotide LAR, respectively.

(**B**) Among patients with CS, doses were reported at treatment initiation and first escalation for 29 and 0 patients who transitioned to lanreotide, and 6 patients and 1 patient who transitioned to octreotide LAR, respectively.

The inner and outer circles show the doses at initiation and after first escalation during the switched treatment. The percentages present the proportion of patients with the reported dose among (**A**) the overall cohorts (58 switched treatment on lanreotide and 7 switched treatment on octreotide LAR) and (**B**) the CS subgroups (29 switched treatment on lanreotide and 6 switched treatment on octreotide LAR).

Abbreviations: CS, carcinoid syndrome; LAR, long-acting release.

REFERENCES

1. American Cancer Society. Treating gastrointestinal carcinoid tumors. 2018. Accessed May 15, 2023. https://www.cancer.org/cancer/types/gastrointestinal-carcinoid-tumor/treating.html

2. American Cancer Society. Treating pancreatic neuroendocrine tumors. 2018. Accessed May 15, 2023. https://www.cancer.org/cancer/types/pancreatic-neuroendocrine-tumor/treating.html

3. Huynh L, Cai B, Cheng M, et al. Analysis of real-world treatment patterns, healthcare resource utilization, and costs between octreotide and lanreotide among patients with neuroendocrine tumors. *Pancreas*. 2019;48(9):1126-1135.

4. Reidy-Lagunes D, Raj N, Saltz L. Commentary: Somatostatin analogs – how we choose, and why. *Semin Oncol.* 2017;44(2):157-158.

5. Nadkarni A, McMorrow D, Patel C, Fowler R, Smith D. Incidence of dose escalation and impact on biologic costs among patients with rheumatoid arthritis treated with three intravenous agents. *J Comp Eff Res.* 2017;6(8):671-682.
